# TSLP Production Induced by Poly(I:C) Stimulation Increased in the Presence of Th2 Cytokines in Patients with Severe ECRS

**DOI:** 10.1055/s-0045-1809159

**Published:** 2025-09-10

**Authors:** Akiko Inoue, Yuriko Tanaka, Hidehito Matsui, Akira Fukuo, Motonari Kondo, Kota Wada

**Affiliations:** 1 Department of Otorhinolaryngology, Medical Center, Toho University Omori, Tokyo, Japan; 2 Department of Otolaryngology (Omori), School of Medicine, Toho University, Tokyo, Japan; 3 Department of Molecular Immunology, School of Medicine, Toho University, Tokyo, Japan

**Keywords:** thymic stromal lymphopoietin, Th2 cytokine, eosinophilic chronic rhinosinusitis

## Abstract

**Introduction:**

In patients with eosinophilic chronic rhinosinusitis (ECRS), viral infection of the upper respiratory tract tends to exacerbate the symptoms.

**Objective:**

To clarify the effect of the cytokines during viral infection in ECRS patients, we investigated the production of thymic stromal lymphopoietin (TSLP) in the sinus mucosa of ECRS patients in the presence of polyinosinic: polycytidylic acid, or poly(I:C), which mimics viral infection, with or without interleukin-4 (IL-4) or IL-13.

**Methods:**

The ECRS patients were classified into mild, moderate, and severe groups based on the scoring system of the Japanese Epidemiological Study of Refractory Eosinophilic Chronic Rhinosinusitis (JESREC). We obtained paranasal sinus mucosa from patients with ECRS through endoscopic sinus surgery, as well as nasal epithelial cells. The cells were stimulated with poly(I:C) in the presence or absence of IL-4 or IL-13. The TSLP concentrations in the culture supernatants were measured using enzyme-linked immunosorbent assay (ELISA) after 24 hours of stimulation.

**Results:**

Nasal epithelial cells from patients with ECRS or healthy controls produced TSLP upon stimulation with poly(I:C) alone, and the addition of IL-4 or IL-13 increased TSLP production. Notably, the increase in poly(I:C)-induced TSLP production from nasal epithelial cells in the presence of IL-4 or IL-13 was greater among the severe group compared to the other groups.

**Conclusion:**

In the sinus mucosa of patients with severe ECRS, TSLP production was enhanced more when poly(I:C) stimulation was combined with IL-4 or IL-13 stimulation. Thus, the Th2-skewed condition of the sinus mucosa during viral infection in patients with more severe ECRS may accelerate disease exacerbation.

## Introduction

Chronic rhinosinusitis (CRS), characterized by nasal discharge, postnasal drip, and nasal congestion, is commonly encountered in the otorhinolaryngological practice. 1 Since 2012, CRS has been classified into two subsets: CRS without nasal polyps (CRSsNP) and CRS with NPs (CRSwNP). 1 Patients with CRSsNP respond well to treatment; however, the prognosis for most patients with CRSwNP remains poor. 1 Infiltration of eosinophils is typically observed in the NPs of patients with CRSwNP in Western countries; however, immune cells other than eosinophils, such as neutrophils and lymphocytes, often infiltrate the NPs of patients with CRSwNP in East Asia. 2 3 4 5 Therefore, ethnicity may influence the CRS phenotype. To distinguish between cases of CRS with a poor or good response to treatment in Japan, an East Asian country, eosinophilic CRS (ECRS) has been proposed as a refractory type based on diagnostic criteria, including computed tomography (CT) findings, presence of NPs, and percentage of eosinophils in the peripheral blood. 6 Although the etiology of CRS remains largely unknown, the categorization of CRS into ECRS and non-ECRS for outcome prediction is considered a reasonable approach to select CRS treatment in Japan. 6 Recently, even in Europe, the type-2 immune response has been emphasized, as well as the phenotype that is the presence or absence of polyps. 7 The concept of “one airway, one disease,” which was proposed in 1997 27 , is adopted to several cases in the otorhinolaryngological practice. For example, the severity of allergic rhinitis and the proportion of patients with a poor prognosis of asthma are correlated in Japan. 8 Thus, it is possible that eosinophils are involved in ECRS pathogenesis, as is the case with airway epithelial cells in patients with asthma. Therefore, we hypothesized that allergic airway diseases share a common etiology.

In addition, epithelial cells and mucosa in the airway are commonly stimulated through pattern recognition receptors (PRRs), such as toll-like receptors (TLRs), upon viral infection, which exacerbates asthma. 9 10 11 Most of the viruses that cause respiratory-tract infections are RNA viruses. Once viral infection occurs, double-stranded (ds) RNAs (dsRNAs) are generated as replication intermediates of the viral genome. 12 These dsRNAs, as well as polyinosinic:polycytidylic acid, or poly(I:C), in experimental settings, are recognized by TLR-3, a member of the TLR family of PRRs. 12 13 Epithelial cells activated by PRRs secrete cytokines that promote the activation of dendritic cells such as thymic stromal lymphopoietin (TSLP), which play an important role in allergic inflammation. 14 Dendritic cells activated by TSLP promote the differentiation of Th2 cells from naïve CD4 ^+^ T cells. 15 Thymic stromal lymphopoietin also plays a role in maintaining a Th2-dominant environment because memory Th2 cells can proliferate in response to TSLP. 14 The expression of the Th2 cytokine interleukin-13 (IL-13) is associated with the recruitment of eosinophils to the airway in response to allergen challenge in patients with asthma. 16 17 Another Th2 cytokine, IL-4, plays a fundamental role in allergic responses by inducing Th2 cells and initiating the production of immunoglobulin E (IgE) in B cells. 18 19 20 However, it remains unclear whether and, if so, how Th2 cytokines are involved in ECRS pathogenesis. To address this issue, using sinus surgical specimens, we investigated the level of TSLP production in cultured epithelial cells from the paranasal sinus mucosa of patients with ECRS with varying severities in the presence and absence of Th2 cytokines and PRR activation.

## Methods

### Patients

**Fig. 1:**
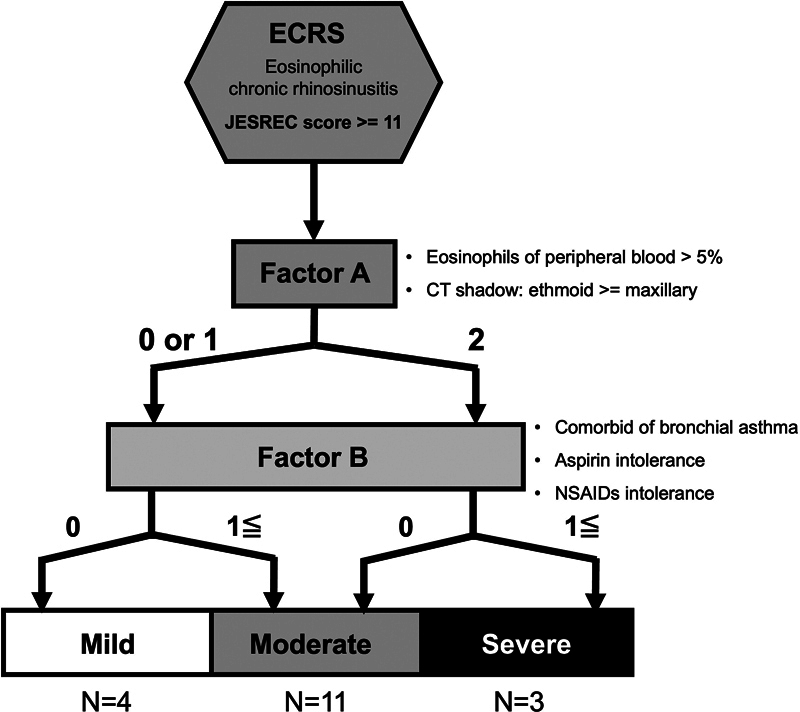
Diagram of eosinophilic chronic rhinosinusitis (ECRS) severity. This classification was adopted from Tokunaga et al. ^6^ Factor A comprises > 5% of eosinophils in the peripheral blood and an ethmoid-dominant shadow on computed tomography, whereas factor B is comorbid (bronchial asthma, aspirin intolerance, intolerance to non-steroidal anti-inflammatory drugs). Factor A ^2^ : both factors are applied (0 or 1); at least one factor is not applied. Factor B (1≦): at least one factor is applied, (0): none of three factors are applied. The numbers presented under the ECRS severity indicate the number of specimens used.

All the patients included provided written informed consent, and the study was approved by the institutional Ethics Committee. The present study included 18 patients with ECRS who underwent surgery at our facility, who were classified according to three degrees of severity based on the JESREC score criteria. 6 As shown in Figure 1 , 4 patients had mild, 11 had moderate, and 3 had severe ECRS. Four healthy control samples were obtained from patients with orbital plate fractures and from those who underwent dacryocystorhinostomy. Patients with other infections (such as HIV, syphilis, and hepatitis B and C), who underwent reoperation, those taking preoperative systemic steroids, those with fungal sinusitis, or subjects with allergic fungal rhinosinusitis were excluded. In total, 22 patients with or without ECRS were enrolled (16 men and 6 women, with a mean age of 56.55 ± 16.744 years) between January 2016 and August 2017.

The preparation of paranasal sinus epithelial cells and stimulation was as follows: the uncinate process of the ethmoid bone was used as a specimen to obtain paranasal sinus epithelial cells. The paranasal sinus mucosa collected during surgery was separated from the bone tissue and fragmented. These fragments were cultured in a collagen I-coated culture dish using bronchial epithelial cell growth medium (BEGM BulletKit [CC-3170], Lonza Group AG) containing penicillin-streptomycin. The paranasal sinus epithelial cells were then stimulated with 25 μg/mL of poly(I:C)) (Sigma-Aldrich) in the presence or absence of 100 ng/mL of recombinant human IL-4 (PeproTech) or 100 ng/mL of recombinant human IL-13 (PeproTech). After 24 hours, the culture supernatant was collected and analyzed for TSLP concentration using the human TSLP enzyme-linked immunosorbent assay (ELISA) Ready-SET-Go! kit (Affymetrix eBioscience). The detection limit was determined to be 8 pg/mL.

### Statistical Analysis:

The IBM SPSS Statistics for Windows software, version 25.0 (IBM Corp.) was used for all analyses. The Wilcoxon signed-rank test was used to examine changes due to differences in stimulation among individuals. The Shapiro-Wilk test was performed to compare patient groups. If a normal distribution was observed, a *t* -test was performed. The correlation analysis was performed using Pearson's product-moment correlation coefficient. Statistical significance was set at *p* < 0.05.

## Results

**Fig. 2:**
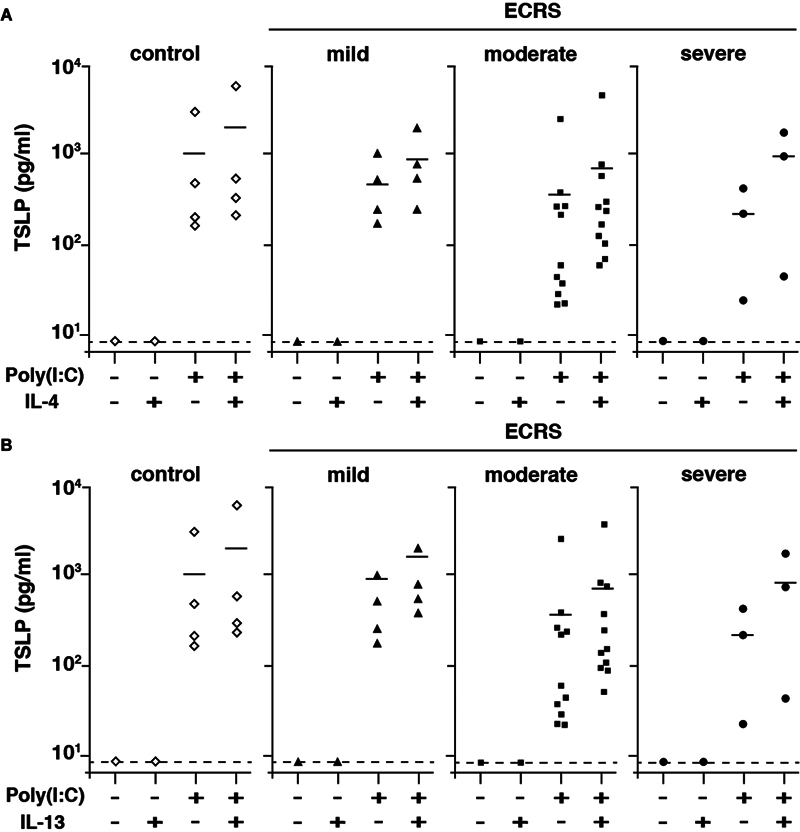
Production of thymic stromal lymphopoietin (TSLP) by paranasal sinus epithelial cells in the presence of polyinosinic: polycytidylic acid, or poly(I:C). Paranasal sinus epithelial cells, classified according to disease severity using the flowchart in Figure 1 , were stimulated with 25 mg/mL of poly(I:C) in the presence or absence of 100 ng/mL recombinant human interleukin-4 (rhIL-4) ( **A** ) or 100 ng/mL of rhIL-13 ( **B** ) for 24 hours. The TSLP protein in the culture supernatant was analyzed through enzyme-linked immunosorbent assay.

We classified the patients with ECRS into 3 groups, as shown in Figure 1 , based on the clinical scoring system from the Japanese Epidemiological Survey of Refractory Eosinophilic Chronic Rhinosinusitis (JESREC). 6 Kim et al. 21 reported the upregulation of Th2 mediators such as Th2 cytokines and TSLP in the epithelial cells of patients with moderate or severe ECRS. Based on these observations, we hypothesized that TSLP is involved in the pathogenesis and progression of ECRS. To clarify whether TSLP is induced in virus-infected paranasal sinus epithelial cells, we obtained primary nasal epithelial cells from the paranasal sinus mucosa of patients with ECRS and healthy controls. To mimic viral infection, we used poly(I:C), which can activate innate immunity via TLR3. We cultured these cells in the presence or absence of poly(I:C) and found that the paranasal sinus epithelial cells of both patients with ECRS and healthy controls produced TSLP in the presence of poly(I:C) ( Fig. 2 ). Although these cells did not produce TSLP in the culture with IL-4 or IL-13, these Th2 cytokines enhanced TSLP production from the paranasal sinus epithelial cells in the stimulation with poly(I:C) ( Fig. 2 ). Therefore, viral infection appears to be the primary inducer of TSLP production in paranasal sinus epithelial cells. In addition, the poly(I:C)-stimulated paranasal sinus epithelial cells produced increased the levels of TSLP in the presence of either IL-4 ( Fig. 2A ) or IL-13 ( Fig. 2B ).

**Fig. 3:**
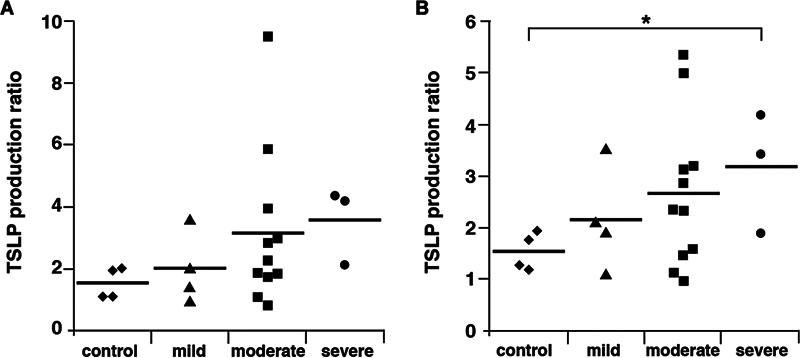
Increase in TSLP production by paranasal sinus epithelial cells in the presence of poly(I:C) with IL-4 or IL-13 compared with poly(I:C) stimulation alone. The rate of increase in TSLP production by paranasal sinus epithelial cells stimulated with poly(I:C) alone or with poly(I:C) combined with IL-13 ( **A** ) or IL-4 ( **B** ) in control, mild ECRS, moderate ECRS, or severe ECRS groups (* *p* < 0.05).

**Fig. 4:**
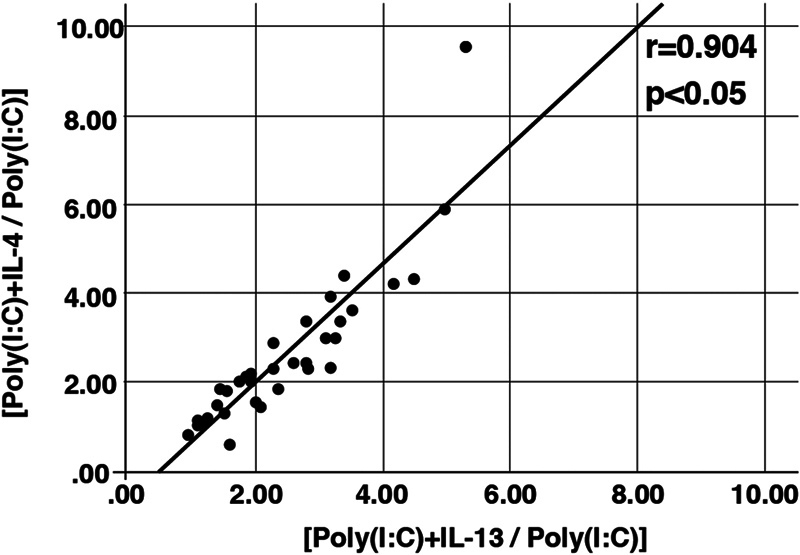
Correlation between the ratio of TSLP production by paranasal sinus epithelial cells in the presence of poly(I:C) and IL-4 or IL-13, compared with stimulation with poly(I:C) alone. The ratio of TSLP production by paranasal sinus epithelial cells stimulated with poly(I:C) combined with IL-4 was compared with the ratio of TSLP production in paranasal sinus epithelial cells stimulated with poly(I:C) combined with IL-13 (r = 0.904; *p* < 0.001 by the Pearson's correlation coefficient).

To investigate the relationship between TSLP production and ECRS severity, we compared the ratio of increase in TSLP production in paranasal sinus epithelial cells stimulated with poly(I:C) alone or with poly(I:C) combined with IL-4 or IL-13. As shown in Figure 3 , the increase in TSLP production in all patients with varying degrees of ECRS severity was greater than among the controls. Particularly, the increase in TSLP production by paranasal sinus epithelial cells from patients with severe ECRS stimulated with poly(I:C) combined with IL-13 was significantly greater than that of the controls ( Fig. 3A ; *t* -test; *p* = 0.042). Although the culture with IL-4 showed a trend similar to that observed in the culture with IL-13, the difference in TSLP production ratio with IL-4 was not statistically significant ( Fig. 3B ). However, a correlation was observed between the TSLP production ratios after IL-4 and IL-13 stimulation ( Fig. 4 ). Accordingly, both IL-4 and IL-13 can enhance poly(I:C)-induced TSLP production by paranasal sinus epithelial cells in patients with severe ECRS more than in those with mild or moderate ECRS. Therefore, we propose that Th2 cytokine-rich paranasal conditions exacerbate ECRS following viral infection, especially in patients with severe ECRS.

## Discussion

Various cell types can respond to TSLP, resulting in the development of Th2-dominant responses that may lead to allergic diseases. 21 Therefore, understanding how TSLP production is regulated is crucial to elucidate the pathogenicity of allergic diseases. In the present study, we showed that TSLP was produced by epithelial cells derived from the paranasal sinus mucosa in response to poly(I:C). The secretion of TLSP induced by poly(I:C) was enhanced in the presence of Th2-type cytokines IL-4 or IL-13. Importantly, the epithelial cells from patients with severe ECRS responded to Th2 cytokines with a greater increase in TSLP production than those from patients with moderate or mild ECRS. Therefore, more severe TSLP-Th2 exacerbating cycles may be initiated in patients with severe ECRS following viral infection.

Bronchial asthma and allergies to nonsteroidal anti-inflammatory drugs influence the severity of ECRS. 6 Therefore, an allergic or Th2-skewed background may affect ECRS pathogenesis. Since TSLP is a key factor in initiating and maintaining Th2-skewed conditions, 22 the ability to produce abundant TSLP in epithelial cells in severe ECRS patients seems reasonable. In addition, the frequency of patients with severe ECRS and a clinical history of asthma is much higher than that of other patients. 6 Therefore, patients with severe ECRS may have Th2-dominant status. This may be a result of epigenetic modifications of the genome in the epithelial cells of patients with severe ECRS under Th2-skewed conditions. Another possibility is the presence of a specific single nucleotide variation (SNV) that may affect *TSLP* gene expression. Several SNVs in the *TSLP* gene have been identified and suggested to be involved in asthma susceptibility across multiple ethnic backgrounds. 23 From the perspective of the “one airway, one disease” concept, a shared genetic factor possibly influences TSLP promoter activity, thereby leading to airway disorders and ECRS. 24

Similar to asthma, respiratory-tract infections in patients with severe ECRS may cause symptoms of exacerbated sinusitis, based on the results of the current study. One possible reason for this observation is that, in patients with severe ECRS, TSLP production is markedly increased in the paranasal sinus mucosa, where Th2 predominance is likely when combined with viral infection. In the current study, cultured epithelial cells did not produce TSLP in the absence of stimulation. Therefore, in the ECRS-affected area of the paranasal sinus, constant stimuli that induce TSLP production likely exists in epithelial cells and potentially in other types of cells. Currently, dupilumab, an anti-IL-4α/IL-13α receptor antibody, 25 is used for the treatment of CRSwNP in Japan. In addition, tezepelumab, an anti-TSLP antibody, 26 is currently in phase-III trials for CRSwNP, which is part of the ECRS in the United States. Accordingly, our results suggest that the combination of dupilumab and tezepelumab might be more effective for the treatment of severe ECRS than the individual use of each drug.

### Limitations

Since the number of samples was limited in the present study, a trend towards a correlation between poly(I:C)-induced TSLP production rate with or without Th2 cytokines and ECRS severity was not statistically proven ( Fig. 3 ). Therefore, although the ratio of TSLP production by paranasal sinus epithelial cells stimulated with poly(I:C) in the presence or absence of IL-13 was statistically different compared with the controls, the mild and moderate ECRS groups might exhibit further distinctions if additional samples are analyzed.

## Conclusion

In the sinus mucosa of patients with severer ECRS, TSLP production was increased when poly(I:C) stimulation was combined with IL-4 or IL-13 stimulation. Thus, the Th2-skewed condition of the sinus mucosa and enhanced immune responses due to viral infection in patients with more severe ECRS may accelerate disease exacerbation.
